# Macrophages in head and neck squamous cell carcinoma: A bibliometric analysis

**DOI:** 10.1097/MD.0000000000036649

**Published:** 2023-12-15

**Authors:** Sai Liang, Ji Wang, Zhaolei Ma, Ming Yu, Zheng-Peng Gong

**Affiliations:** a Department of Clinical Medicine, Guizhou Medical University, Guiyang, China; b Key Laboratory of Endemic and Ethnic Diseases, Ministry of Education & Key Laboratory of Medical Molecular Biology of Guizhou Province, Guizhou Medical University, Guiyang, China; c Department of Geriatrics, The Affiliated Hospital of Guizhou Medical University, Guiyang, China; d Department of Otorhinolaryngology Head and Neck Surgery, Affiliated Hospital of Guizhou Medical University, Guiyang, China.

**Keywords:** bibliometrics, Citespace, head and neck squamous cell carcinoma, tumor-associated macrophages, VOSviewer

## Abstract

**Introduction:**

The tumor microenvironment in head and neck squamous cell carcinoma (HNSCC) is densely infiltrated by macrophages. Utilizing bibliometric analysis, the characteristics, hotspots for research, and research frontiers related to macrophages in HNSCC were reviewed.

**Methods:**

The Web of Science Core Collection database was queried for relevant articles published from 2000 to 2022. VOSviewer and CiteSpace software were utilized to evaluate and visualize macrophage-related HNSCC research trends and hotspots.

**Results:**

Assessment of original articles revealed that the annual number of publications regarding the role of macrophages in HNSCC has increased steadily over the past 23 years. China produced the most articles, whereas the United States had the highest number of citations and highest H-index. Wuhan University and Oral Oncology were the most productive affiliation and journal, respectively. The paper published by Bray et al in the CA-A Cancer Journal for Clinicians in 2018 had the greatest number of citations. The keywords “expression,” “cancer,” and “tumor-associated macrophages (TAMs)” occurred most frequently.

**Conclusions:**

This bibliometric investigation discovered that publications about macrophages in HNSCC are steadily increasing. The majority of studies focused on macrophage polarization, macrophage markers, and inflammation in the tumor microenvironment. Furthermore, our bibliometric analysis revealed that the immunosuppressive role of tumor-associated macrophages in the tumor microenvironment and resistance to therapy in HNSCC have recently received attention.

## 1. Introduction

Head and neck cancer is the seventh most prevalent form of cancer worldwide, with squamous cell carcinoma of the head and neck accounting for over 90% of cases.^[1,2]^ Head and neck squamous cell carcinoma (HNSCC), which includes malignancies of the oral cavity, oropharynx, nasopharynx, larynx, and hypopharynx, accounted for 878,348 new cases of cancer and 444,347 cancer-related deaths worldwide.^[3,4]^ Despite the wide use of surgery, radiotherapy, and chemotherapy, the 5-year survival rate of patients with HNSCC remains at 50%, and the local risk of recurrence can approach as high as 50% and the rate of distant metastasis can reach 25%.^[5–7]^ The mechanisms underlying the poor clinical outcomes for patients with HNSCC require further investigation.

HNSCC tissue has fewer lymphocytes, fewer natural killer (NK) cells, and different antigens present than normal tissues, and is considered a “cold tumor.” HNSCC can evade immune monitoring by a variety of means.^[8]^ Tumor tissues are more than aggregates of proliferating tumor cells; they are complex tissues comprised of a variety of cell types that together create the tumor microenvironment (TME), producing a complicated network that promotes tumor development.^[9]^ Macrophages are a type of innate myeloid cell that develops from circulating bone marrow-derived monocytic progenitors and are critical components of the innate immune response.^[10]^ Tumor-associated macrophages (TAMs), are a common kind of leukocyte found in the TME, and the link between innate and adaptive immunity play key roles in angiogenesis, proliferation, and metastasis.^[11]^

HNSCC recruits macrophages, which can have an immunosuppressive effect. The presence of TAMs is linked to poor prognosis in HNSCC.^[12]^ Studies related to the recruitment, survival, repolarization, and phagocytosis of TAMs have highlighted new techniques for regressing malignant tumors, including HNSCC. These techniques inhibit the immunosuppressive function of TAMs and/or activate the potential pro-inflammatory capabilities of TAMs, eliciting an adaptive immune response and, ultimately, improving anticancer immune responses.^[11]^ Due to the close relationship between HNSCC and macrophages, TAMs may become an important target in the field of HNSCC treatment in the future. Research on this topic has attracted great interest. Literature analysis helps scholars to understand the development of HNSCC-associated macrophages and reveals the evolutionary trends in this field. Therefore, it is necessary to review and summarize the relevant reports on this topic in a structured manner and predict the future trends and hotspots related to TAMs in HNSCC.

Bibliometrics is the study of library and information science through quantitative analysis of bibliographic materials.^[13]^ It is a revolutionary tool for statistically evaluating the impact of extant research on certain research fields periods, countries/regions, research collaborations, journals, institutions, and authors.^[14]^ Bibliometric analysis is a current, intuitive, and unbiased technique to follow advancements and study information.^[12]^ VOSviewer is a software tool for displaying and studying web-based maps. CiteSpace is Java-based bibliometric mapping program. VOSviewer and CiteSpace are used in healthcare, sports management, and urban planning.^[15,16]^ Bibliometric analysis has been extensively utilized in a variety of study fields.^[13,17]^ By evaluating databases and literary features, bibliometrics provides a useful way to estimate developing trends in a scientific field and revealing primary research paths. In addition, bibliometrics can guide experimental tactics and financing decisions with solid evidence.^[18]^ In the present bibliometric review, we identify research hotspots and future trends regarding macrophages in HNSCC by analyzing published research from the last 23 years.

## 2. Materials and methods

### 2.1. Data sources and search strategies

**Figure 1.**
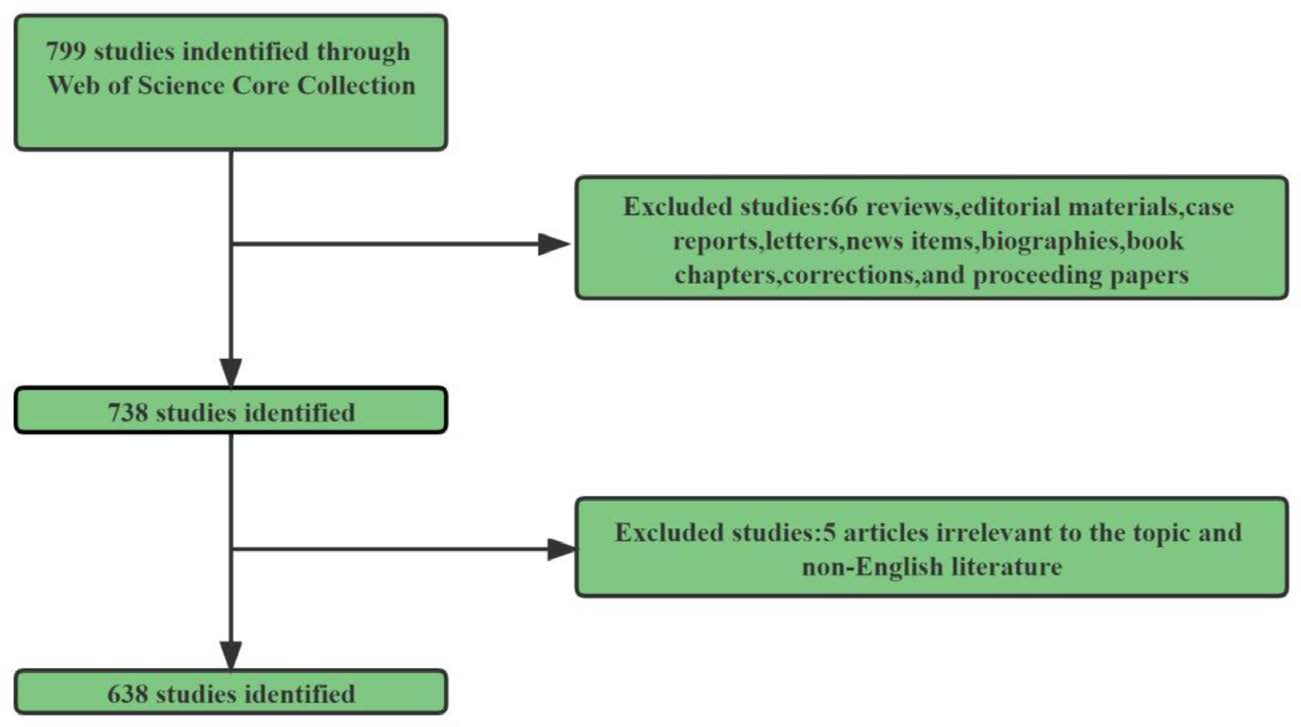
Flowchart of the search strategy.

For bibliometric analysis, the Science Citation Index-Expanded and Social Sciences Citation Index of the Web of Science Core Collection were utilized. To avoid deviations, literature retrieval was conducted on a single day (January 30, 2023) in consideration of the rapid database renewal. The publication window for this study spans from 2000 to 2022. TS= (“macrophage” OR “macrophages”) AND TS = (“head and neck carcinoma” or “head and neck cancer” or “head and neck Squamous cell carcinoma” or “HNSCC” or “Oral squamous cell carcinoma” or “OSCC” or “laryngeal carcinoma” or “hypopharyngeal carcinoma”) constituted the search terms. Only original articles written in English from various publication types were included. Two researchers (SL and JW) conducted the primary data search independently, and after discussing any potential discrepancies, the final agreement reached 0.90, indicating substantial concordance.^[19]^ In the end, 638 articles were analyzed for this study. Figure 1 depicts the detailed evaluation workflow. Two researchers independently conducted the data analysis. To reach a consensus, disagreements were resolved through consultation or by enlisting the aid of external specialists. We recorded the titles, abstracts, keywords, countries, institutions, journals, authors, references, and citations for each article.

### 2.2. Data analysis methods

All of the Web of Science Core Collection (WoSCC) data were converted into text and then input into the bibliometric software for analysis. The bibliometric online analysis platform (http://bibliometric.com), VOSviewer 1.6.18 (Leiden University, Leiden, Netherlands), and CiteSpace 6.1.R6, 64-bit (Drexel University, Philadelphia, PA) were utilized to identify countries, institutions, journals, authors, co-cited authors, keywords, references, and network characteristics of keyword bursts, and to visually present the results.

### 2.3. Bibliometric analysis

The number of publications and citations that were often utilized to represent the bibliographic material were bibliometric indicators. The H-index connects productivity and effect by determining the threshold that connects the number of publications and the number of citations. In other words, if a researcher authored H publications, each of which was cited at least H times, she or he would have a H-index.^[20]^ Although the H-index was designed to evaluate individual academic success, it might also be used to describe the publication output of a country or region, an institution, or a journal. Furthermore, the impact factor determined from the most recent version of Journal Citation Reports is widely regarded as one of the most important measurements of the quality and influence of medical journals.^[21]^

VOSviewer can be applied to build scientifically grounded knowledge networks that demonstrate the progress of research areas, inter-institutional cooperation, and future research hotspots. In this analysis, we used VOSviewer to visually assess term co-occurrence and create density maps. Co-occurrence analysis in VOSviewer can group keywords into multiple clusters, with each cluster denoted by a distinctive color. Through a term co-occurrence network, cluster analysis of research hotspots may be enhanced to visualize and detect the trend of development. Concurrently, VOSviewer is utilized to illustrate the relationship between authors and co-cited authors.

We analyzed the identified literature in series using CiteSpace software, including countries, institutions, co-cited references, and characteristics of keyword bursts, to survey the hotspots of HNSCC Macrophage-related research. The nodes on the created network visual map represented the objects analyzed, with larger nodes representing objects with a high frequency. Furthermore, we performed a centrality analysis using the CiteSpace software, which is an index that defines the significance of network nodes, with higher centrality representing a more prominent node. The concept of centrality is used to quantify the significance of a node’s position in the network. The greater the centrality, the greater the number of network connections that pass through that node.^[22]^

## 3. Results

### 3.1. Annual growth trend in publication output

**Figure 2.**
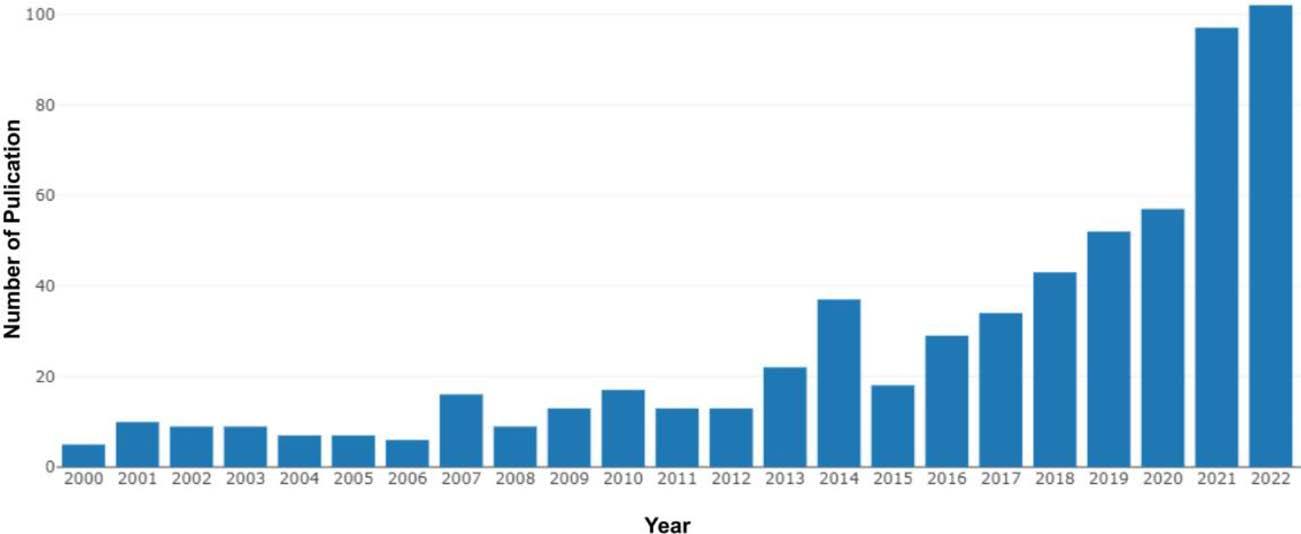
Trends in the number of publications on HNSCC macrophage-related research from 2000 to 2022.

From 2000 to 2022, 638 original articles matching our search terms were published. Between 2000 and 2022, macrophage-related HNSCC studies showed an overall increasing trend (Fig. 2). Since 2015, the number of publications on HNSCC macrophages has expanded considerably, with more than 5 times as many publications in 2022 compared to 2015. From 2015 to 2022, macrophage research activity at HNSCC reached its zenith, with 432 papers published in 8 years, representing more than half of the total.

### 3.2. Distribution of countries, regions, and institutions

**Figure 3.**
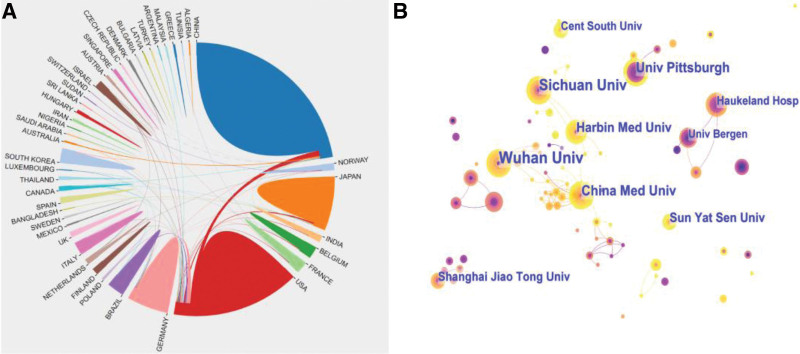
Cooperation between countries/regions (A) and institutions (B) which contributed to publications on macrophages in HNSCC from 2000 to 2022. The color and thickness of a node’s inner ring indicate the frequency of occurrence over time.

The network of national cooperation for HNSCC macrophage research is depicted in Figure 3A. Table 1 lists the top ten contributing countries. China contributed the most (232), followed by the United States (152), Japan (85), and Germany (59). China was the leading contributor to HNSCC macrophage research among the top 10 nations. The H- index measures the significance of network nodes. The US had the highest H-index (35), followed by China (32), and Germany (32). Centrality research found that the United States (0.77), followed by Japan (0.25), and Germany, comprised the network’s core (0.25). In a collaborative network, more centrality corresponded to increased cooperation.

**Table 1:** Publications in the 10 most productive countries/regions in the field of macrophages in HNSCC.

Rank	Country/region	NP	NC	Centrality	H-index
1	Peoples R China	232	3411	0.1	32
2	USA	152	5756	0.77	39
3	Japan	85	2312	0.25	28
4	Germany	59	1929	0.25	25
5	China Taiwan	26	736	0	16
6	Brazil	23	514	0.07	12
7	India	20	302	0	9
8	France	17	609	0.09	13
9	Norway	17	281	0.13	10
10	South Korea	16	379	0.09	9

HNSCC = head and neck squamous cell carcinoma, NC = number of citations, NP = number of publications.

Figure 3B depicts the network of institutional collaboration, highlighting the top ten collaborating institutions, which include Wuhan University (25), Sichuan University (18), China Medical University (16), the University of Pittsburgh (15), and Harbin Medical University (14) (Table 2). The top centrality rankings were held by China Medical University (0.02) and Sichuan University (0.01).

**Table 2:** Ranking of top 10 institutions in the field of macrophages in HNSCC from 2000 to 2022.

Rank	Institutions	Country	NP	NC	Centrality	H-index
1	Wuhan University	China	25	632	0.00	14
2	Sichuan University	China	18	911	0.01	13
3	China Medical University	China	16	208	0.02	12
4	University of Pittsburgh	USA	15	809	0.00	13
5	Harbin Medical University	China	14	173	0.00	10
6	Sun Yat-Sen University	China	14	386	0.00	10
7	Shanghai Jiao Tong University	China	12	300	0.00	8
8	Center South University	China	12	258	0.00	9
9	Haukeland University	Norway	12	124	0.00	8
10	University of Bergen	Norway	12	64	0.00	9

HNSCC = head and neck squamous cell carcinoma, NC = number of citations, NP = number of publications.

### 3.3. Contributions of authors and co-cited authors

**Figure 4.**
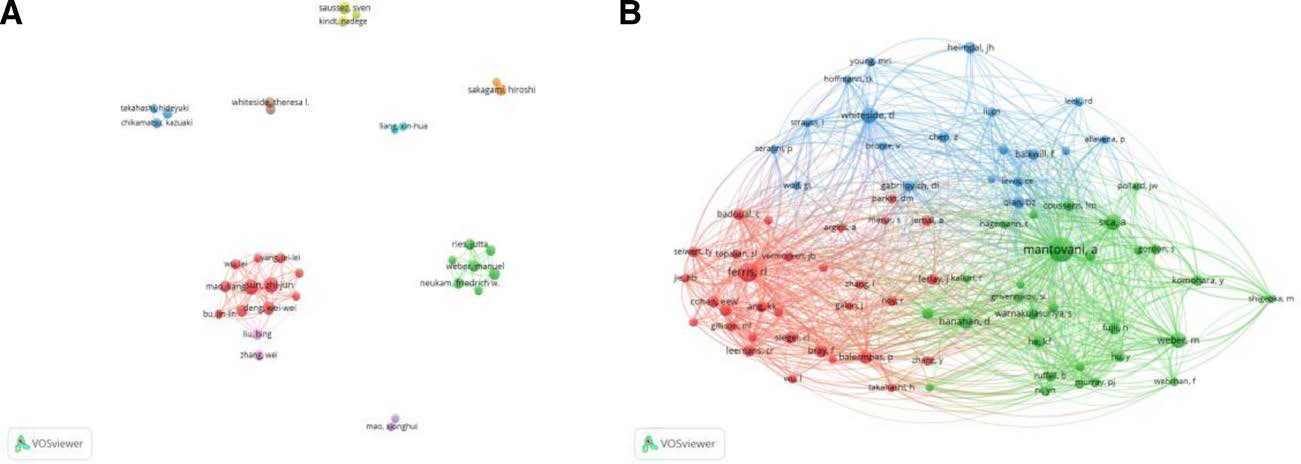
A map of the author (A) and the cited author (B) in macrophage research in HNSCC spanning 2000 to 2022.

The top 10 productive authors are listed in Table 3. They contributed 112 publications, accounting for 17.5% of the total number of papers. Sun ZJ. (Wuhan University, China) led the field investigating macrophages in HNSCC with 14 publications, followed by Zhang, WF. (Wuhan University, China), and Deng WW (Wuhan University, China) (Fig. 4A).

**Table 3:** Top 10 authors and co-cited authors in the field of macrophages in HNSCC.

Rank	Author	Count	Centrality	Co-cited author	Co-citation	Centrality
1	Sun,ZJ	14	0.01	Mantovani, A	118	0.18
2	Zhang, WF	13	0.02	Ferris, RL	70	0.07
3	Deng,WW	10	0.01	Sica, A	60	0.03
4	Mao, L	8	0.01	Whiteside, TL	46	0.16
5	Yu,GT	8	0.00	Hanahan, D	45	0.02
6	Weber,M	8	0.01	Fujii, N	43	0.05
7	Wehrhan,F	8	0.00	Weber, M	42	0.02
8	Sakagami,H	8	0.00	He, KF	42	0.07
9	Ries,J	8	0.01	Siegel, RL	41	0.00
10	Neukam,FW	8	0.01	Bray, F	41	0.01

HNSCC = head and neck squamous cell carcinoma.

The most productive research groups and possible partners can be found with the help of a visual map of coauthored papers, which can also assist in the formation of cooperative ties between scientists. In Figure 4B, we can see the results of a VOSviewer visualization of authors who have received at least 20 citations. There are 79 circles on the map, each one representing a different author. As a result of name overlap, it may be impossible to read all of the names. Open squares represent writers who are actively working together on a study. The co-cited author with the greatest number of co-citations was Mantovani, A. from the Azienda Ospedaliera Universitaria Integrata of Verona, with the highest centrality of 0.18.

### 3.4. Analysis of journals and co-cited journals

Table 4 depicts the characteristics of the 10 most active journals and the 10 most co-cited journals on the topic of macrophages in HNSCC. *Oral Oncology, Frontiers in Oncology*, and the *Journal of Oral Pathology & Medicine* published the most articles on HNSCC macrophage research. *Cancer Research*, which has a high impact factor (13.312), has also published a large number of articles on HNSCC macrophage research. *Cancer Research* had the most co-citations (1149) among the co-cited journals, followed by *Clinical Cancer Research* (868 co-citations) and *Oral Oncology* (727 co-citations).

**Table 4:** Top 10 journals and co-cited journals in the field of macrophages in HNSCC.

Rank	Journal	Count	IF (2021)	JCR	Co-Cited Journals	Co-citation	IF (2021)	JCR
1	Oral Oncology	26	5.972	Q1	Cancer Research	1149	13.312	Q1
2	Frontiers in Oncology	22	5.738	Q2	Clinical Cancer Research	868	13.801	Q1
3	Journal of Oral Pathology & Medicine	17	3.539	Q2	Oral Oncology	727	5.972	Q1
4	Oncotarget	14	NA	NA	Journal of Immunology	592	5.426	Q1
5	Head and Neck-Journal for the Sciences and Specialties of the Head and Neck	14	3.821	Q1	International Journal of Cancer	512	7.316	Q1
6	International Journal of Cancer	13	7.316	Q1	PLoS One	497	3.752	Q2
7	Cancers	12	6.575	Q1	Nature	459	69.504	Q1
8	Cancer Research	10	13.312	Q1	Journal of Clinical Oncology	439	50.717	Q1
9	Anticancer Research	9	2.435	Q4	Proceedings of the National Academy of Sciences of the United States of America	399	12.779	Q1
10	BMC Cancer	8	4.638	Q2	Oncotarget	396	NA	NA

HNSCC = head and neck squamous cell carcinoma, IF = impact factor, JCR = Journal Citation Reports.

### 3.5. Analysis of co-cited references and reference burst

**Figure 5.**
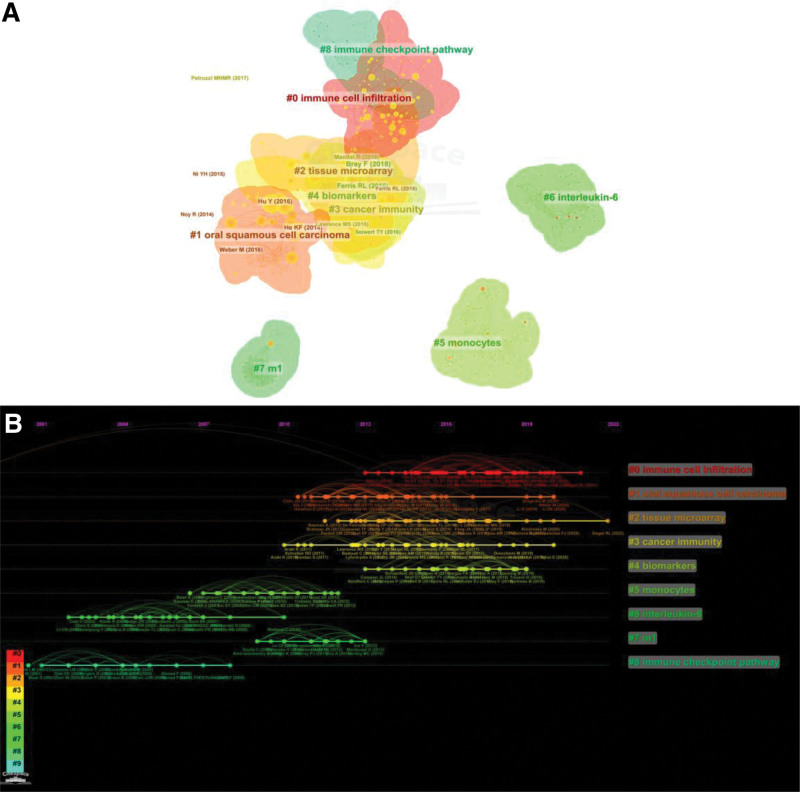
A literature map of HNSCC macrophage-related research from 2001 to 2022 (A). Each node’s diameter is proportional to the total number of citations for all relevant articles. Co-cited clusters with timeline labels (B). This view shows the appearance and time of 9 clusters.

In this study, the co-citation relevance of 832 cited references from 638 publications was examined to generate a cluster network graph (Fig. 5A). Each node corresponds to a referenced article. The links between the nodes represent the frequency of citations of the same article. The diameter of a node is proportional to the total number of co-cited articles. There were 9 primary clusters of co-cited references, including oral squamous cell carcinoma, tissue microarray, cancer immunity, biomarkers, monocytes, interleukin-6, m1, and immune checkpoint pathway. Figure 5B depicts a chronological perspective of the clustering diagram, which supports the findings of developing research hotspots in HNSCC macrophage research. Table 5 provides a list of the 10 most cited papers. Bray, F. (2018) was the top cited author in *CA-A Cancer Journal for Clinicians* (43 citations), followed by Ferris, R.L. (2016) in the *New England Journal of Medicine* (42 citations) and Hu, Y. (2016) in the *Journal of Experimental & Clinical Cancer Research* (31 citations).

**Table 5:** Top 10 co-cited references in the field of macrophages in HNSCC from 2000 to 2022.

Rank	Author	Title	Journal	IF (2021)	Centrality	Co-Citation
1	Bray F (2018)	Global cancer statistics 2018: GLOBOCAN estimates of incidence and mortality worldwide for 36 cancers in 185 countries	CA-A Cancer Journal for Clinicians	286.130	0	43
2	Ferris RL (2016)	Nivolumab for Recurrent Squamous-Cell Carcinoma of the Head and Neck	New England Journal of Medicine	176.079	0.04	42
3	Hu Y (2016)	Tumor-associated macrophages correlate with the clinicopathological features and poor outcomes via inducing epithelial to mesenchymal transition in oral squamous cell carcinoma	Journal of Experimental & Clinical Cancer Research	12.658	0.04	31
4	He KF (2014)	CD163 + tumor-associated macrophages correlated with poor prognosis and cancer stem cells in oral squamous cell carcinoma	Biomed Research International	3.246	0.06	27
5	Mandal R (2016)	The head and neck cancer immune landscape and its immunotherapeutic implications	JCI Insight	9.484	0.02	22
6	Weber (2016)	Prognostic significance of macrophage polarization in early stage oral squamous cell carcinomas	Oral Oncology	5.972	0.02	21
7	Ni YH (2015)	Microlocalization of CD68 + tumor-associated macrophages in tumor stroma correlated with poor clinical outcomes in oral squamous cell carcinoma patients	Tumor Biology	NA	0.02	20
8	Ferris RL (2015)	Immunology and Immunotherapy of Head and Neck Cancer	Journal of Clinical Oncology	50.717	0.01	19
9	Petruzzi MNMR (2017)	Role of tumor-associated macrophages in oral squamous cells carcinoma progression: an update on current knowledge	Diagnostic Pathology	3.196	0.01	18
10	Lawrence MS (2015)	Comprehensive genomic characterization of head and neck squamous cell carcinomas	Nature	69.504	0.05	18

HNSCC = head and neck squamous cell carcinoma, IF = impact factor.

**Figure 6.**
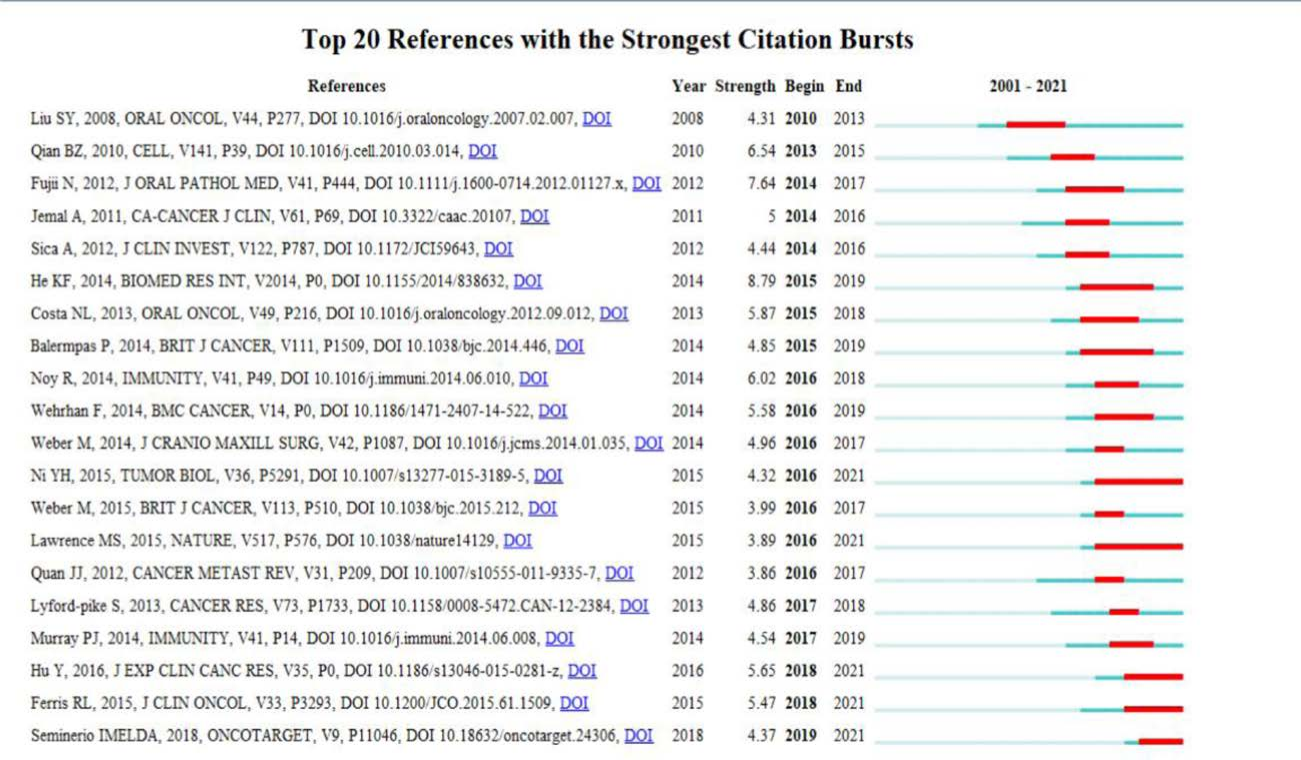
Visualization map of the top 20 references in macrophage-related HNSCC with the highest citation bursts.

The burst detection algorithm is an effective data analysis tool for capturing dramatic increases in the popularity of references or keywords over a certain period. This function identifies concepts or themes that are discussed publicly during a given period. The burst detection algorithm was used in this study to extract key references and keywords for macrophage-related HNSCC research. Figure 6 depicts the top 20 references with the most powerful citation bursts. The blue lines in this graph represent the period, and the red lines represent the time frame in which the reference burst occurred. Among these references, the article written by Bray, F.^[23]^ is referenced with the strongest burst value (13.02).

### 3.6. Keyword co-occurrence cluster analysis concerning research hot spot

**Figure 7.**
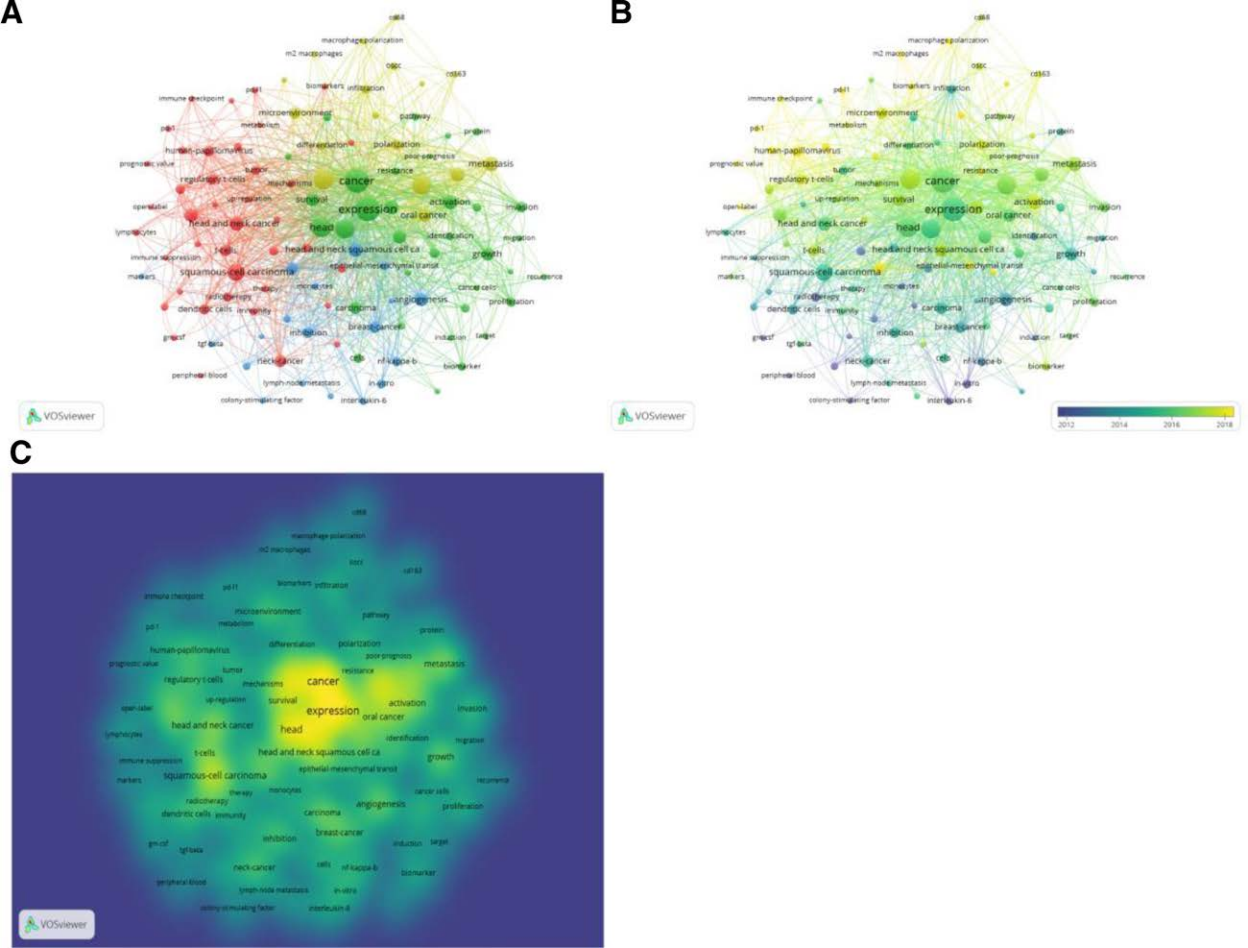
(A) A mapping of keywords in macrophage-related HNSCC research field. (B) A distribution of keywords based on average frequency of occurrence. (C) Frequency evolution of keywords.

VOSviewer analysis was used to search the titles and abstracts of the 638 articles retrieved for keywords. Then, a map was produced with 110 terms (out of a total of 2735 keywords) with at least 10 occurrences per phrase, which were grouped into 4 clusters. Cluster 1 (in red) in Figure 7A was primarily concerned with the role of macrophages in HNSCC. Cluster 2 (in green) and cluster 3 (in blue) reflected primarily the cellular processes and molecular pathways of macrophage response to HNSCC. Cluster 4 (in yellow) was concerned with macrophage microenvironment alterations in HNSCC. The keywords with the highest frequency on the map were expression (175), cancer (170), and tumor-associated macrophages (120). As shown in Figure 7B, VOSviewer split the colors of all keywords according to their average publication year. We also evaluated keyword frequency evolution in a density plot (Fig. 7C). The greater the density, the closer the hue is to yellow. Research hotspots in a given field are typically located in regions with higher grayscale levels.

**Figure 8.**
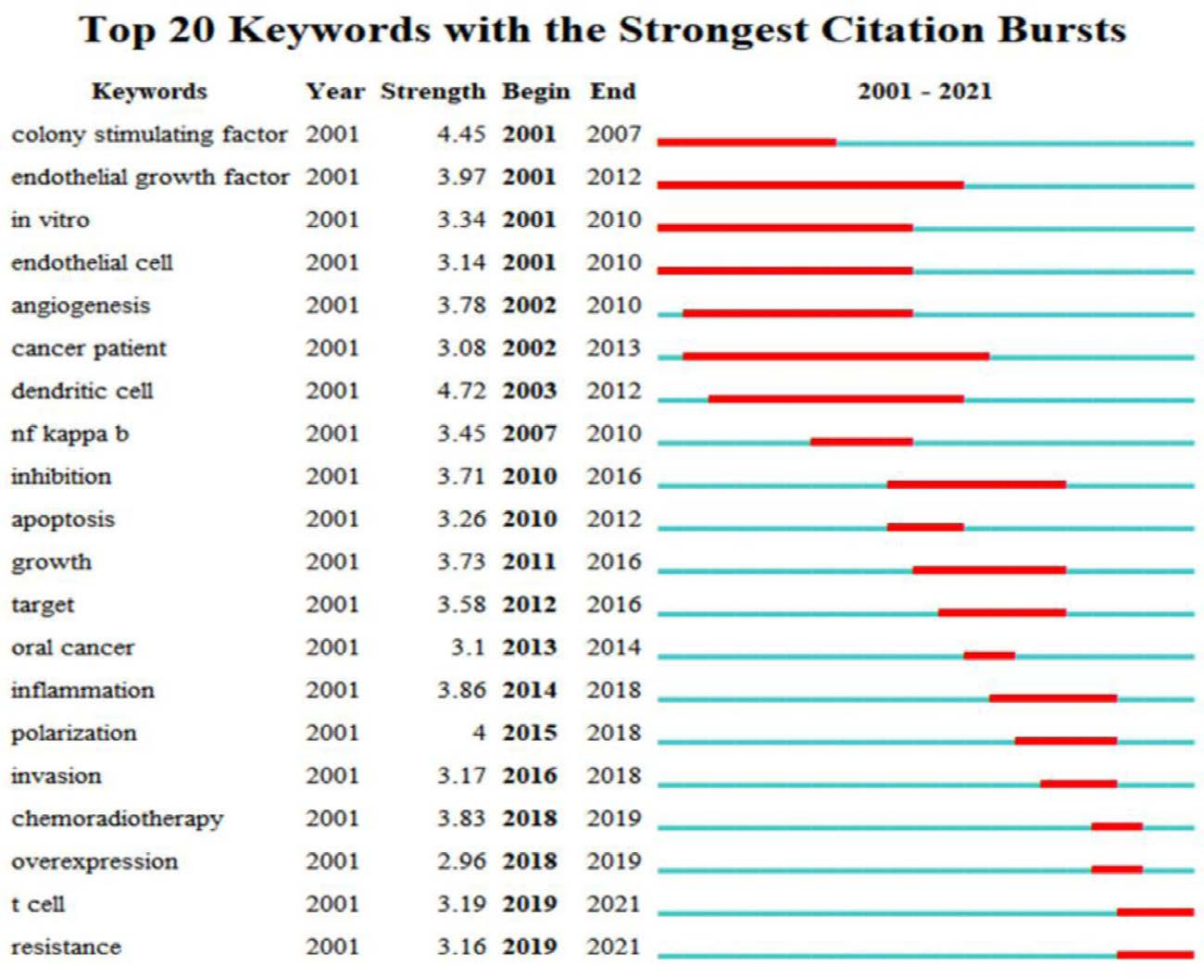
Visualization map of the top 20 keywords with the strongest citation bursts in macrophage-related HNSCC.

From this keyword analysis of the 638 articles included in the WoSCC database, keyword outbreaks from 2000 to 2022 were determined. In Figure 8, a blue line slicing through the years represents the chronology, while a red line illustrates the burst period, indicating the starting and finishing years, as well as the citation burst duration. To concentrate on keywords that represented trends in HNSCC Macrophages research, we omitted terms with little or no research value. From 2000 to 2022, the term “dendritic cell” had the greatest burst strength (4.72), followed by “colony stimulating factor” (4.45), “polarization” (4), “endothelial growth factor” (3.97), and “inflammation” (3.86).

## 4. Discussion

Using Bibliometrics, VOSviewer, and Citespace software, we performed a bibliometric analysis to evaluate the developing trends and hotspots related to macrophages in HNSCC research from the Science Citation Index-Expanded and Social Sciences Citation Index of the Web of Science Core Collection. We gathered 638 unique papers and reviews that were published between 2000 and 2022. According our assessment, the annual number of publications had a general rising trend, with the rate of growth accelerating after 2015. The significant expansion in the annual number of publications was primarily attributable to ground-breaking papers with high local citation scores.

We found that China ranks first among the top countries/regions in terms of the number of publications on macrophages in HNSCC, indicating that China is a highly productive nation in this research field. Seven Chinese institutions ranked among the top 10 affiliations for macrophage research in HNSCC, indicating that China had the world’s most productive institutions in this field, which largely explains China’s quick development in this sector over the past decade. In contrast to China, the United States had a relatively high centrality and H-index, and the United States has advanced further in this field than the rest of the world. Japan and Germany both produced a large number of publications and had relatively high centrality scores. This suggests that, if Chinese academics and institutions aim to be more impactful than institutions in other nations on this subject, they should improve the quality of their articles on this topic to increase the impact of their research. Country-based research networks show a low density, indicating that research groups are largely independent, and all institutions have low centrality, suggesting a lack of inter-institutional communication. This emphasizes the need for more collaboration on this topic.

We found that Sun, Z.J. has published the most papers in the field of macrophage-related HNSCC research. The top 5 most productive authors in this field over the last 21 years were Zhang, W.F., Deng, W.W., Mao, L., and Yu, G.T. Zhang, W.F. was the most productive author with a high centrality score (0.02). Nonetheless, it is worth noting that, globally, researchers in macrophage-related HNSCC comprise a distinct geographical profile, with the majority of these scholars based in Europe and the United States. These authors typically work in otolaryngology departments at university-affiliated hospitals. We speculate that improving communication and collaboration among global researchers would improve the production of macrophage-related HNSCC research.

The most published journals in the field of macrophages in HNSCC include Oral Oncology, Frontiers in Oncology, Journal of Oral Pathology & Medicine, Oncotarget, Head and Neck-Journal for the Sciences and Specialties of the Head and Neck, International Journal of Cancer, Cancers, Cancer Research, Anticancer Research, and BMC Cancer. These journals have had a considerable impact on head and neck cancer research all over the world, as well as have shaped direction of research in their respective scientific domains. The top ten co-cited references from 2000 to 2022 indicated that researchers are focused on tumor-associated macrophages. Furthermore, the article “Global cancer statistics 2018: GLOBOCAN estimates of incidence and mortality worldwide for 36 cancers in 185 countries” published by Bray, F.^[23]^ was a landmark article and has the highest co-citation rate of the articles we analyzed.

Analysis of keyword co-occurrence provides information on the distribution and evolution of various research hotspots within a given field. We found that research into macrophage markers, macrophages in inflammation, macrophage polarization, immune checkpoints, and poor-prognosis have been hotspots in HNSCC research in recent years. Keywords may also include commonly utilized macrophage-surface markers, such as CD68, CD80, and CD163. CD80, and CD163 are markers for M1 and M2 macrophages, respectively, whereas CD68 is a general macrophage marker.^[24]^ The CD163/CD68 ratio is used to assess M2 polarization and M2 polarization of macrophages in tumors has been linked to metastatic disease and poor prognosis.^[25,26]^ As shown in Figure 7A, inflammatory studies related to HNSCC, the role of TAMs in the invasion, metastasis, angiogenesis, and immunosuppression of HNSCC, and treatments targeting TAMs have been widely studied. TME inflammation is associated with leukocyte infiltration and the expression of proinflammatory cytokines and chemokines, including IL-1, IL-6, CCL2, CCL5, CXCL8, CD40L, and TNF.^[27,28]^ Inflammation results in rapid cell proliferation^[29]^ and, in conjunction with DNA-damaging growth factors and inducible nitric oxide synthase, can facilitate development and hasten the progression of cancer.^[30]^ Even though most TAMs produce the anti-inflammatory marker IL-10, this does not affect the pro-inflammatory nature of the TME because it diverts the ability of cytotoxic T-cells to respond to malignant cells.^[11,29,30]^

The effects of the tumor microenvironment on macrophage polarization has garnered a growing amount of attention. Cancer cells in the TME control the polarization of TAMs directly, and TAMs predominantly exhibit the antitumor M1 phenotype during the earliest stages of tumor growth.^[27]^ M1 TAMs exhibit robust antigen presentation ability, can help CD8 + T and NK cells multiply through IL-6, IL-12, and TNF, and can boost the cytotoxicity of CD8 + T and NK cells to kill tumor cells.^[31–33]^TAMs are dynamic and comprise an assortment of macrophage subtypes, and the ratio of phenotypes and subtypes changes as cancer progresses.^[34]^ Tumor cells can secrete CSF-1, which binds to the CSF-1 receptor on the surface of TAMs, causing them to polarize to M2 TAMs. M2 TAMs promote tumor growth, metastasis, and angiogenesis, and contribute to tumor immune escape. The presence of M2 TAMs is associated with poor prognosis.^[35,36]^ TAMs overexpress PD-L1 and CTLA-4 in HNSCC, which inhibits the TCR-signal pathway and encourages the development of an immunosuppressive TME.^[37]^ There is a correlation between high PD-L1 levels and the presence of CD68 + and CD163 + TAMs in oral squamous cell carcinoma.^[38]^ PD-L1 binds to PD-1 receptors on the surface of activated T cells and sends inhibitory signals to T cells, allowing tumor cells to escape from the immune system.^[39]^ Malignant transformation of HNSCC is characterized by local invasion and distant metastasis.^[40]^ Epithelial–mesenchymal transition is typical of invasion and metastasis of HNSCC.^[41]^ TAMs can reduce the expression of E-cadherin and induce extensive epithelial–mesenchymal transition in HNSCC cancer cells by secreting bioactive substances^[42,43]^ which promote HNSCC invasion and metastasis.^[44]^ However, tumor invasion and metastasis are intricate processes, and the mechanisms by which TAMs promote the invasion and metastasis of HNSCC remains to be fully elucidated.

Keywords with a burst indicate emerging trends and research frontiers (Fig. 8). There are 4 prominent emerging areas of macrophage research in HNSCC: chemoradiotherapy (2018–2019), overexpression (2018–2019), T cell (2019–2021), and resistance (2019–2021). Chemoradiotherapy (CRT) continues to be one of the most often used cancer treatments, and new research suggests that it is most successful when an antitumoral immune response is generated. On the other hand, CRT has been shown to promote immunosuppressive pathways that must be stopped or reversed to maximize the immune-boosting effects of CRT.^[45,46]^ One of the most difficult tasks in modern medicine is improving anticancer responses to existing therapies. Chemotherapy resistance is often associated with higher levels of IL-6 and prostaglandin E2, both of which are inflammatory mediators that lead to the development of M2 macrophages.^[47]^ M2 macrophages are more resistant to radiation therapy than M1 macrophages, making cancer treatment difficult because most TAMs are M2. M2-phenotype TAMs increase the number of M2-like macrophages in the TME after treatment with antiangiogenic agents.^[48]^ Learning about the function and mechanism of M2 TAMs in the TME is essential for more fully understanding malignant transformation and developing therapies to prevent it, and converting M2 macrophages to an M1 phenotype is a promising anticancer therapeutic strategy.^[49]^

TAMs are indispensable immune cells in the HNSCC microenvironment, and the number of M2 macrophages increases steadily with tumor growth. The number of TAMs is negatively correlated with the prognosis of HNSCC. TAMs support invasion, metastasis, angiogenesis, and immunosuppression of HNSCC through the production and release of a variety of growth factors, cytokines, chemokines, and proteolytic hydrolases, thereby promoting the HNSCC progression. TAMs play an important role in the tumor microenvironment and possess antitumor activity, which may be a potential target for the future treatment of HNSCC. Current therapeutic strategies for targeting TAMs in HNSCC include (1) reducing the total number of TAMs; (2) decreasing the recruitment of TAMs at the primary tumor site; (3) inhibiting the activation of TAMs; and (4) reprogramming M2-like macrophages to a tumor-killing M1-like phenotype.^[50,51]^ Currently, there are many research targets related to TAMs, including colony stimulating factor 1, Pexidartinib, Sotuletinib, Emactuzumab, and so on.^[52,53]^ Despite the increasing research on TAMs, there are still many unknowns about their role in the genesis, regulation, and treatment of HNSCC, which need to be explored by more studies. In conclusion, TAMs are an attractive target for HNSCC. In the future, it may become an innovative anticancer approach for the treatment of HNSCC.

## 5. Limitations

Based on bibliometrics and visual analysis, this study provides significant evidence regarding the current research status and the general trend of academic frontiers in the field of macrophages in HNSCC. This data was extracted from the WoSCC database; therefore, studies not collected in WoSCC are not included. Due to the inability of VOSviewer to analyze the full publication text, some details may be omitted. Due to the latency in publication, some recently published papers that are meritorious and impactful, but have low citation counts, may have been excluded from our analysis. In addition, variations in databases that are continually updated may result in discrepancies between search results and the actual number of publications included. To conduct a more precise literature analysis, bibliometrics software’s knowledge graph should be combined with specific relevant literature. Nonetheless, we believe this bibliometric report accurately describes the current overall direction and general trends emerging in the field of macrophages in HNSCC.

## 6. Conclusions

The literature on macrophage-related HNSCC was reviewed using bibliometrics in this study. We show how macrophage-related HNSCC publications and citations have changed over the last 23 years. The number of articles discussing macrophage-related HNSCC is growing. In HNSCC, clinical studies or clinical guidelines published in high-impact journals are frequently cited. Future research will continue to focus on macrophage polarization, modifications in the macrophage-associated tumor microenvironment, exploration of mechanisms of action of immunotherapy, and studies on macrophage-mediated treatment resistance. We believe that this bibliometric analysis will be useful to researchers to improve their understanding of the state of research in macrophage-related HNSCC.

## Author contributions

**Conceptualization:** Sai Liang, Ji Wang, Zhengpeng Gong.

**Data curation:** Sai Liang, Ming Yu, Zhaolei Ma.

**Funding acquisition:** Ming Yu.

**Investigation:** Zhaolei Ma.

**Methodology:** Zhaolei Ma, Zhengpeng Gong.

**Project administration:** Ming Yu, Zhengpeng Gong.

**Resources:** Zhengpeng Gong.

**Software:** Sai Liang, Ji Wang.

**Supervision:** Zhaolei Ma, Zhengpeng Gong.

**Validation:** Sai Liang, Ji Wang, Ming Yu.

**Visualization:** Sai Liang, Zhaolei Ma.

**Writing – original draft:** Sai Liang, Ji Wang, Zhengpeng Gong.

**Writing – review & editing:** Ming Yu, Zhengpeng Gong.
